# Association of Urinary Iodine Concentration With Cognitive Function Among Older Adults: NHANES 2011–2014

**DOI:** 10.1002/fsn3.70906

**Published:** 2025-09-03

**Authors:** Zeyong Sun, Fengyang Song, Xiaojuan Wei, Kehua Liao, Lu Lu, Shaozhou Mo, Wentan Huang

**Affiliations:** ^1^ Department of Nuclear Medicine People's Hospital of Guangxi Zhuang Autonomous Region Nanning China

**Keywords:** cognitive function, iodine nutrition, NHANES, urinary iodine concentration

## Abstract

The purpose of this study is to investigate the relationship between urinary iodine concentrations (UIC) and cognitive function among elderly individuals in certain regions of the United States, addressing a significant gap in the existing literature regarding urinary iodine and cognitive decline in older populations. UIC and cognitive function assessments of participants in the National Health and Nutrition Examination Survey were selected for the 2011–2014 cycle. Cognitive function assessments included: (1) a word learning and recall module from the Coalition to Establish a Registry for Alzheimer's Disease (CERAD); (2) an animal fluency test; and (3) the Digit Symbol Substitution Test (DSST). Based on the average z‐scores of three cognitive tests, participants were classified into low cognitive status and normal cognitive status, with those in the lowest 25th percentile of average z‐scores categorized as having low cognitive performance. Iodine deficiency (UIC < 100 μg/L) was associated with a significantly reduced risk of cognitive decline compared to the adequate iodine intake group (UIC 100–199 μg/L) (adjusted OR = 0.54, 95% CI: 0.29–0.99; *p* = 0.046). No significant differences in risk were observed for above requirement (UIC 200–299 μg/L) or excessive iodine intake (UIC ≥ 300 μg/L). This cross‐sectional study provides the first evidence linking UIC to cognitive outcomes in older adults. Contrary to expectations, participants with iodine deficiency demonstrated significantly lower risk of cognitive decline compared to those with adequate iodine intake.

## Graphical abstract

This study investigated the association between urinary iodine concentration and cognitive function in older adults using NHANES 2011–2014 data. Our study found that iodine deficiency (UIC < 100 μg/L) was associated with a significantly reduced risk of cognitive decline compared to the adequate iodine intake group (UIC 100–199 μg/L) (adjusted OR = 0.54, 95% CI: 0.29–0.99; *p* = 0.046). No significant differences in risk were observed for above requirement (UIC 200–299 μg/L) or excessive iodine intake (UIC ≥ 300 μg/L).

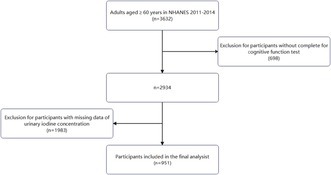

## 1 Introduction

With advancements in medical technology and improvements in living standards, life expectancy has steadily increased worldwide. This trend is accompanied by a growing risk of cognitive decline among the elderly. According to 2021 statistics, approximately 6.9 million Americans aged 65 and older were diagnosed with Alzheimer's disease in 2020, and this number is expected to rise to 13.8 million by 2060 (Rajan et al. 2021). Alzheimer's disease has become one of the major health threats to older adults, ranking as the fifth leading cause of death among Americans aged 65 and older (Association As 2024). While available drugs target the biological mechanisms of Alzheimer's disease, achieving better treatment outcomes still depends heavily on optimizing lifestyle factors (Association As 2023; Scheltens et al. 2021). Therefore, exploring lifestyle factors related to cognitive function has become crucial for improving patient prognosis and effectively preventing the disease.

Iodine is a vital trace element in the human diet, primarily obtained through the consumption of iodine‐rich foods or iodized salt. Our iodine nutritional status is commonly assessed by measuring urinary iodine concentration (UIC) (World Health Organization 2007). Iodine is essential for the development and function of several brain structures, especially the hippocampus, as well as brain microstructures like myelin and neurotransmitters (Andersson et al. 2012; Schroeder and Privalsky 2014; Abel et al. 2017).

The importance of iodine in neurodevelopment throughout various life stages is well established. During pregnancy, mild iodine deficiency (ID) can lead to impaired cognitive function in offspring (Bath et al. 2013; Hynes et al. 2013). Two rigorously designed, randomized, double‐blind, placebo‐controlled studies have shown a link between iodine deficiency and cognitive function in school‐aged children (Redman et al. 2016). Another study found that children aged 6–16 years with a UIC below 100 μg/L had lower intelligence quotient (IQ) scores compared to those with a UIC above 100 μg/L (Santiago‐Fernandez et al. 2004). Studies have shown that adults in communities with severe iodine deficiency exhibit apathy and sluggishness (Hetzel 1983). Unfortunately, evidence linking iodine nutrition to cognitive function in older adults is scarce. Therefore, this cross‐sectional study aims to investigate the relationship between UIC and cognitive function among older adults in the United States, providing insights into the potential impact of iodine nutritional status on cognitive health in the elderly and addressing a significant gap in the existing literature regarding urinary iodine and cognitive function in older populations.

## 2 Methods

The National Health and Nutrition Examination Surveys (NHANES) has been carried out as an ongoing survey since 1999, with data being published biennially. Further information on the sampling methodology and the data utilized in this study can be found on the NHANES website (https://www.cdc.gov/nchs/nhanes). The NHANES protocol received approval from the Research Ethics Review Board of the National Center for Health Statistics (NCHS), and informed consent was obtained from all participants.

**FIGURE 1:**
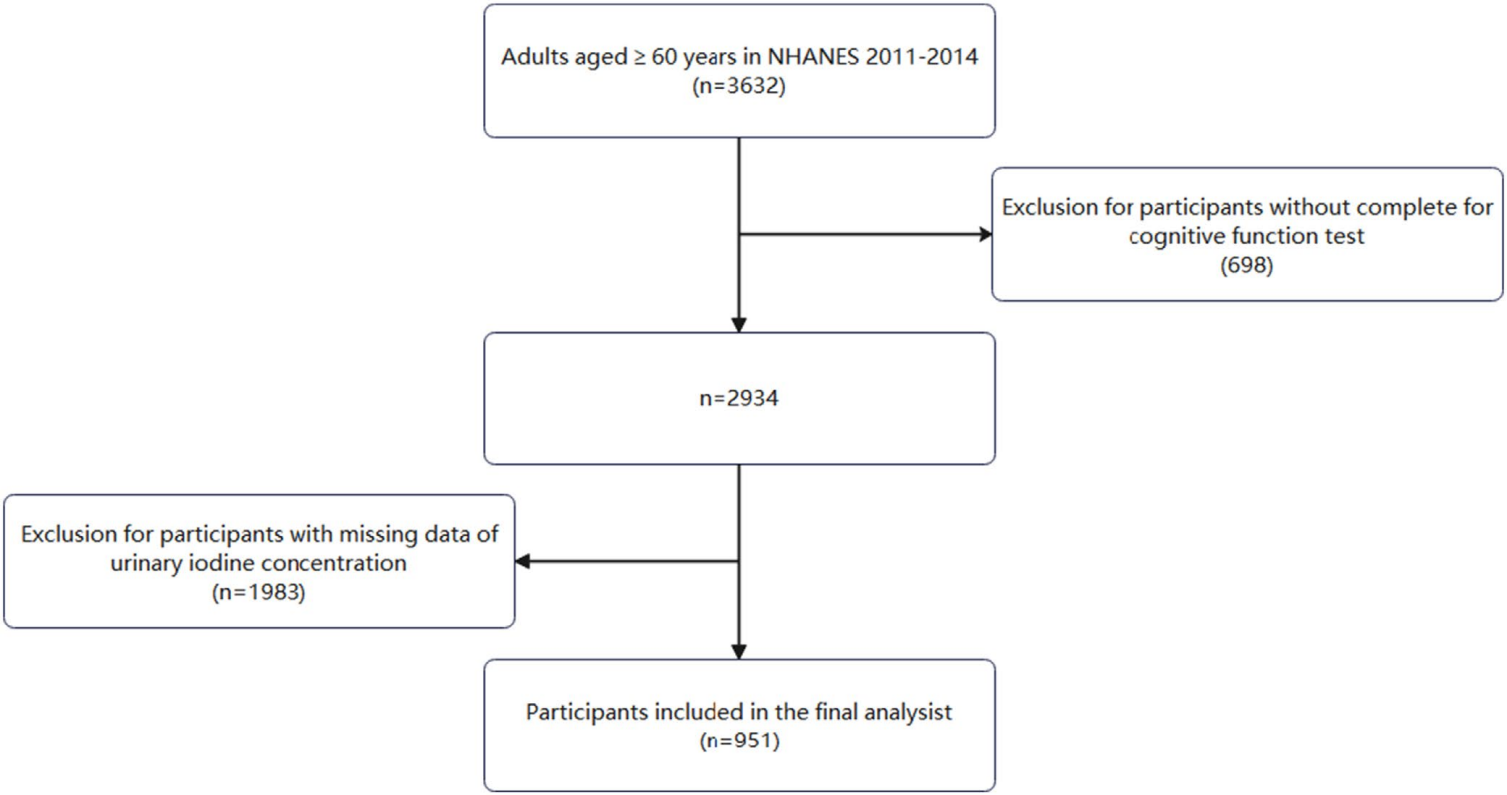
Inclusion and exclusion flowchart.

In this cross‐sectional study, we included 3632 individuals ≥ 60 years from two NHANES cycles (2011–2012 and 2013–2014). We excluded subjects without UIC measurements (*n* = 1983) and those with incomplete cognitive function assessments (*n* = 698). Additionally, multiple imputations were performed for subjects with missing covariate information. A total of 951 participants were eventually included in the study for analysis (Figure 1).

### 2.1 UIC Measurement

In NHANES, UIC is determined by ICP‐DRC‐MS (Inductively Coupled Plasma Dynamic Reaction Cell Mass Spectrometry). The laboratory's equipment, procedures, and location remained consistent across the two cycles. Comprehensive guidelines for sample collection and processing are available on the NHANES website.

### 2.2 Cognitive Assessment

In NHANES, individuals aged 60 years or more could take part in the cognitive function assessment. Cognitive function was assessed using: (1) a word learning and recall task from the Coalition to Establish a Registry for Alzheimer's Disease (CERAD); (2) an animal fluency task; and (3) the Digit Symbol Substitution Test (DSST).

#### 2.2.1 A Brief Description of Each Assessment Is Provided

The CERAD Word Learning subtest (CERAD W‐L) evaluates both immediate and delayed verbal memory performance (Strauss et al. 2006). It involves three consecutive learning trials, each presenting 10 unrelated words that the participant reads aloud. Immediately after the word presentation, the participant is asked to recall as many words as possible, with the order of words changing across trials. Each trial is scored out of 10, and the immediate recall score (CERAD‐IR), which is the total sum of the three trials, ranges from 0 to 30. Delayed recall (CERAD‐DR), scored from 0 to 10, occurs approximately 8–10 min after the word learning trials, following two other cognitive tests: Animal Fluency and DSST.

The Animal Fluency test evaluates verbal fluency, related to executive function (Strauss et al. 2006). Research has shown its ability to distinguish individuals with normal cognition from those with slight cognitive impairment or advanced disorders, such as Alzheimer's disease (Henry et al. 2004; Clark et al. 2009; Canning et al. 2004). This test is frequently employed in large‐scale screenings and epidemiological studies (Clark et al. 2009; Canning et al. 2004; Grundman et al. 2004). During the test, participants are instructed to name as many animals as possible within 60 s, receiving one point for every animal mentioned.

The DSST, a component of the Wechsler Adult Intelligence Scale (WAIS III), measures processing speed, attention span, and working memory (Wechsler 1997). The test is commonly employed in large‐scale screenings, epidemiological investigations, and clinical studies (Bienias et al. 2003; Plassman et al. 2007; Proust‐Lima et al. 2007). It includes a printed version with a key at the top, linking 9 numbers to their respective symbols. Participants have 2 min to match the numbers to the symbols in 133 boxes. The score reflects the total number of correct matches. For detailed scoring information, see the 2011–2012 and 2013–2014 NHANES CFQ questionnaire data files.

#### 2.2.2 Criteria for Cognitive Performance

Participants were grouped into low and normal cognitive status based on the average z‐scores from three cognitive tests. Those with an average z‐score within the lowest 25th percentile were classified as having low cognitive performance. This dichotomous approach has been used in previous national survey studies, including NHANES (Blaum et al. 2002; Brody et al. 2019). Based on previous research (Brody et al. 2019), individuals in the lowest 25th percentile likely included those with cognitive impairments due to aging, dementia, delirium, or those who consistently remained in this range throughout their lives. The 25th percentile threshold for the global cognitive measure was calculated from the entire analytic sample while accounting for the complex survey design.

### 2.3 Covariates

In addition, based on demographic data, we selected the following covariates for the multivariable logistic model: age (60 to < 70, ≥ 70 years), gender (male, female), race/ethnicity (Non‐Hispanic White, Non‐Hispanic Black, Mexican American, Other Hispanic, Other Race—including Multi‐Racial), body mass index (BMI, defined as weight (kg)/height^2^ (m^2^); normal: < 25 kg/m^2^, overweight: 25 to < 30 kg/m^2^, obese: ≥ 30 kg/m^2^), education level (Low Educational Level: less than 9th grade, 9–11th grade [includes 12th grade with no diploma], and high school graduate/GED or equivalent; Intermediate Educational Level: Some College or AA degree; High Educational Level: College graduate or above), marital status (married, never married, living with partner, other: widowed, divorced, or separated), family poverty income ratio (PIR) (PIR < 1.00: below poverty level; PIR ≥ 1.00: above poverty level), Smoking status was categorized as follows: “no” for individuals who smoked fewer than 100 cigarettes in their lifetime; “yes” for those who smoked more than 100 cigarettes in their lifetime but are not currently smoking, or those who smoked more than 100 cigarettes in their lifetime and either smoke occasionally or daily. Drinking status was classified as: “never” for individuals who had consumed fewer than 12 alcoholic drinks in their lifetime; “former” for those who had 12 or more drinks in a single year but did not drink in the past year, or those who had consumed at least 12 drinks over their lifetime but did not drink in the past year; “current mild alcohol use” for females consuming no more than one drink per day and males consuming no more than two drinks per day; “current moderate alcohol use” for females consuming two or more drinks per day, males consuming three or more drinks per day, or binge drinking on two or more occasions per month; and “current heavy alcohol use” for females consuming three or more drinks per day, males consuming four or more drinks per day, or binge drinking (defined as consuming four or more drinks on one occasion for females, and five or more drinks for males) on five or more days per month. Additionally, we considered other chronic diseases from questionnaires and laboratory data, including hypertension, diabetes, stroke, and thyroid problems (determined by self‐reported physician diagnosis). Variables that can affect cognitive function, as reported by previous literature, include renal insufficiency (Yaffe et al. 2010), which was determined based on self‐reported physician diagnosis, and weekly physical activity time (Tang et al. 2024; Yun et al. 2019), referring to the time spent walking or bicycling, performing tasks around the home or yard, engaging in work activities, and participating in recreational activities.

### 2.4 Statistical Analysis

Categorical variables were presented as percentages (%), while continuous variables were described using the mean with standard deviation (SD) or the median with interquartile range (IQR), based on the data distribution. Group differences were evaluated using one‐way ANOVA for variables with a normal distribution, the Kruskal‐Wallis test for non‐normally distributed data, and the chi‐square test for categorical variables.

According to the 2007 edition of the WHO/UNICEF/IGN guidelines, this study categorizes UIC into four groups to assess the iodine nutritional status of the population: iodine deficiency group (UIC < 100 μg/L), adequate iodine intake group (UIC: 100–199 μg/L), above requirement group (UIC: 200–299 μg/L), and excessive iodine intake group (UIC ≥ 300 μg/L). We used a binary logistic regression model to assess the association between UIC and the risk of low cognitive performance, with adequate iodine intake as the reference. Three models were constructed: the crude model, with no covariate adjustment; Model 1, adjusted solely for socio‐demographic characteristics and individual lifestyle habits (age, gender, race/ethnicity, body mass index, education level, marital status, family poverty income ratio, smoking status, drinking status, weekly physical activity time); and Model 2, fully adjusted to include all covariates. We utilized multiple imputation, conducted over 5 iterations using the chained equations method in the R package ‘*mice*’, to maximize statistical power and minimize potential bias arising from missing data (Park et al. 2017). In Model 2, we further explored the linear and non‐linear relationships between changes in UIC and cognitive performance using restricted cubic splines (RCS).

We performed stratified and interaction analyses to assess whether the association between UIC and low cognitive performance remains consistent across various subgroups. In addition to the findings (Ge et al. 2024), which indicated that the impact of cognitive function may vary by gender and age, we also analyzed race/ethnicity (White vs. non‐White), education level (≤ 12 years vs. > 12 years), BMI (< 25 vs. ≥ 25 kg/m^2^), marital status (married or living with partner vs. living alone: widowed, divorced, or separated), PIR (PIR < 1.00 vs. PIR ≥ 1), drinking status (never vs. former and current), smoking status (never vs. former and current), hypertension (no vs. yes), diabetes (no vs. yes), physical activity time (< 150 min per week vs. ≥ 150 min per week), and thyroid problems (no vs. yes). Subgroup heterogeneity was evaluated using multivariate logistic regression, and interactions between subgroups and UIC were tested through likelihood ratio tests. Following previous research on the influence of alcohol consumption on cognitive function, we performed a sensitivity analysis by excluding participants classified as ‘never’ drinkers (Yen et al. 2022).

All statistical analyses were conducted using R Statistical Software (version 4.2.2, http://www.R‐project.org, R Foundation) and the Free Statistics analysis platform (version 1.9, Beijing, China, http://www.clinicalscientists.cn/freestatistics). Free Statistics is a user‐friendly software that facilitates common statistical analyses and data visualization. It operates with R as the core statistical engine, while its graphical user interface (GUI) is built using Python. A two‐tailed *p* value of less than 0.05 was considered statistically significant.

## 3 Results

### 3.1 Baseline Characteristics

A total of 951 participants were selected for the final data analysis. Based on their cognitive performance (low vs. normal), we presented the baseline characteristics of these participants (Table 1). Overall, 51.3% of the participants were female. Approximately 54.2% of the participants were aged between 60 and 69 years, while 45.8% were aged 70 years or older. Individuals in the low cognitive function group exhibited higher UIC levels and were predominantly male, aged 70 years and older, with lower educational attainment and income levels. They were less likely to drink alcohol, had less physical activity, and were more prone to comorbidities such as stroke, hypertension, and diabetes. No significant differences were observed between the low cognitive function group and the normal cognitive function group in terms of BMI (*p* = 0.553), smoking status (*p* = 0.298), renal failure (*p* = 0.088), and thyroid problems (*p* = 0.540). Baseline characteristics were consistent between the crude data and the five imputed datasets. No significant statistical differences were observed among the covariates across the five imputations (*p* > 0.05; Table S1).

**TABLE 1:** Characteristic of study participants by cognitive function status.

Characteristics	Total (*n* = 951)	Normal cognitive function (*n* = 713)	Low cognitive function (*n* = 238)	*p*
Age, *n* (%)				< 0.001
60–69	515 (54.2)	416 (58.3)	99 (41.6)	
≥ 70	436 (45.8)	297 (41.7)	139 (58.4)	
Gender, *n* (%)				< 0.001
Male	463 (48.7)	320 (44.9)	143 (60.1)	
Female	488 (51.3)	393 (55.1)	95 (39.9)	
Race/ethnicity *n* (%)				< 0.001
Non‐Hispanic White	452 (47.5)	369 (51.8)	83 (34.9)	
Non‐Hispanic Black	225 (23.7)	158 (22.2)	67 (28.2)	
Mexican American	84 (8.8)	55 (7.7)	29 (12.2)	
Other Hispanic	92 (9.7)	56 (7.9)	36 (15.1)	
Other Race‐including Multi‐Racial	98 (10.3)	75 (10.5)	23 (9.7)	
Body mass index, *n* (%)				0.504
< 25 kg/m^2^	265 (28.4)	197 (27.9)	68 (29.7)	
25 < 30 kg/m^2^	333 (35.7)	247 (35)	86 (37.6)	
≥ 30 kg/m^2^	336 (36.0)	261 (37)	75 (32.8)	
Education level, *n* (%)				< 0.001
Low educational level	470 (49.5)	292 (41)	178 (75.1)	
Intermediate educational level	269 (28.3)	233 (32.7)	36 (15.2)	
High educational leve	210 (22.1)	187 (26.3)	23 (9.7)	
Marry status, *n* (%)				0.026
Married	533 (56.2)	419 (58.9)	114 (47.9)	
Never married	56 (5.9)	38 (5.3)	18 (7.6)	
Living with partner	24 (2.5)	18 (2.5)	6 (2.5)	
Widowed, divorced, or separated individuals	336 (35.4)	236 (33.2)	100 (42)	
Poverty income ratio, *n* (%)				< 0.001
< 1	166 (19.3)	98 (15.1)	68 (32.2)	
≥ 1	694 (80.7)	551 (84.9)	143 (67.8)	
Smoking status, *n* (%)				0.139
No	459 (48.3)	354 (49.6)	105 (44.1)	
Yes	492 (51.7)	359 (50.4)	133 (55.9)	
Drinking status *n* (%)				< 0.001
Never	146 (15.6)	106 (15.1)	40 (17.2)	
Former	254 (27.2)	166 (23.7)	88 (37.9)	
Mild	380 (40.7)	312 (44.5)	68 (29.3)	
Moderate	86 (9.2)	74 (10.6)	12 (5.2)	
Heavy	67 (7.2)	43 (6.1)	24 (10.3)	
Hypertension, *n* (%)				0.014
No	288 (30.3)	231 (32.4)	57 (23.9)	
Yes	663 (69.7)	482 (67.6)	181 (76.1)	
Diabetes, *n* (%)				< 0.001
No	641 (67.4)	505 (70.8)	136 (57.1)	
Yes	310 (32.6)	208 (29.2)	102 (42.9)	
Stroke, *n* (%)				0.016
No	896 (94.3)	679 (95.4)	217 (91.2)	
Yes	54 (5.7)	33 (4.6)	21 (8.8)	
Renal insufficiency, *n* (%)				0.088
No	891 (93.9)	673 (94.7)	218 (91.6)	
Yes	58 (6.1)	38 (5.3)	20 (8.4)	
Thyroid problem				0.540
No	824 (86.6)	615 (86.3)	209 (87.8)	
Yes	127 (13.4)	98 (13.7)	29 (12.2)	
UIC (μg/L), median (IQR)	154.2 (84.3, 262.4)	146.3 (81.6, 248.3)	169.1 (102.8, 294.2)	0.014
WPAT (min), median (IQR)	320.0 (140.0, 780.0)	360.0 (150.0, 825.0)	235.0 (120.0, 600.0)	0.030

Abbreviations: min, minute; UIC, urinary iodine concentrations; WPAT, weekly physical activity time.

### 3.2 Relationship Between UIC and Low Cognitive Function

The univariate analysis indicated that factors such as age, gender, race, education level, marital status, drinking status, hypertension, diabetes, PIR, stroke, physical activity time, and UIC were all associated with low cognitive function (Table 2).

**TABLE 2:** Univariate association of covariates with low cognitive function.

Variable	OR (95% CI)	*p*	Variable	OR (95% CI)	*p*
Age (years)			Marry status, *n* (%)		
60–69	1 (reference)		Married	1 (reference)	
≥ 70	1.97 (1.46–2.65)	< 0.001	Never married	1.74 (0.96–3.17)	0.069
Gender			Living with partner	1.23 (0.48–3.16)	0.674
Male	1 (reference)		Widowed, divorced, or separated individuals	1.56 (1.14–2.13)	0.005
Female	0.54 (0.4–0.73)	< 0.001	Smoking status, *n* (%)		
Race/ethnicityn			No	1 (reference)	
Non‐Hispanic White	1 (reference)		Yes	1.25 (0.93–1.68)	0.140
Non‐Hispanic Black	1.89 (1.3–2.73)	0.001	Drinking status, *n* (%)		
Mexican American	2.34 (1.41–3.9)	0.001	Never	1 (reference)	
Other Hispanic	2.86 (1.77–4.63)	< 0.001	Former	1.4 (0.9–2.19)	0.135
Other Race‐including Multi‐Racial	1.36 (0.81–2.3)	0.247	Mild	0.58 (0.37–0.9)	0.016
Body mass index, *n* (%)			Moderate	0.43 (0.21–0.87)	0.020
< 25 kg/m^2^	1 (reference)		Heavy	1.48 (0.8–2.74)	0.214
25 < 30 kg/m^2^	1.01 (0.7–1.46)	0.963	Hypertension, *n* (%)		
≥ 30 kg/m^2^	0.83 (0.57–1.21)	0.340	No	1 (reference)	
Education level, *n* (%)			Yes	1.52 (1.09–2.13)	0.014
Low educational level	1 (reference)		Diabetes, *n* (%)		
Intermediate educational level	0.25 (0.17–0.38)	< 0.001	No	1 (reference)	
High educational level	0.2 (0.13–0.32)	< 0.001	Yes	1.82 (1.34–2.47)	< 0.001
Poverty income ratio, *n* (%)			Thyroid problem		
< 1	1 (reference)		No	1 (reference)	
≥ 1	0.37 (0.26–0.54)	< 0.001	Yes	0.87 (0.56–1.36)	0.540
Stroke, *n* (%)			UIC (μg/L)		
No	1 (reference)		Adequate Iodine Intake (100–199)	1 (reference)	
Yes	1.99 (1.13–3.51)	0.017	Iodine Deficient (< 100)	0.63 (0.43–0.93)	0.020
Renal insufficiency, *n* (%)			Above requirement (200–299)	0.99 (0.64–1.54)	0.979
No	1 (reference)		Excessive iodine intake (≥ 300)	1.14 (0.76–1.69)	0.529
Yes	1.62 (0.93–2.85)	0.091	Weekly physical activity time (minute)		
			< 150 min/per week	1 (reference)	
			≥ 150 min/per week	0.65 (0.43–0.99)	0.044

Table 3 displays the findings from the logistic regression analysis. In the unadjusted model, the iodine deficiency group was associated with a lower likelihood of low cognitive function compared to the adequate iodine intake group, with an odds ratio (OR) of 0.63 (95% CI: 0.43–0.93, *p* = 0.020). After adjusting for socio‐demographic characteristics and individual lifestyle factors, the odds ratio remained consistent at 0.54 (95% CI: 0.29–0.97, *p* = 0.041). In the final adjusted model, which accounted for additional potential confounding factors, participants in the iodine deficiency group showed significantly reduced odds of low cognitive performance compared to the adequate iodine intake group, with an adjusted odds ratio of 0.54 (95% CI: 0.29–0.99; *p* = 0.046). The results from the five multiple imputation datasets also showed similar findings (OR < 1), with statistical significance, as detailed in Table S2. It is worth noting that the odds ratios for cognitive impairment in the above requirement and excessive iodine intake groups gradually increased, although the associations were not statistically significant (*p* > 0.05).

**TABLE 3:** Association of UIC and low cognitive performance risk.

Urinary iodine concentration (μg/L)	Cases/participants	Crude		Model 1		Model 2	
		OR (95% CI)	*p*	OR (95% CI)	*p*	OR (95% CI)	*p*
Iodine deficient (< 100)	55/290 (19.0)	0.63 (0.43–0.93)	0.020	0.54 (0.29–0.97)	0.041	0.54 (0.29–0.99)	0.046
Adequate iodine intake (100–199)	86/319 (27.0)	1.00 (reference)		1.00 (reference)		1.00 (reference)	
Above requirement (200–299)	40/149 (26.8)	0.99 (0.64–1.54)	0.979	0.91 (0.46–1.80)	0.786	0.94 (0.47–1.87)	0.856
Excessive iodine intake (≥ 300)	57/193 (29.5)	1.14 (0.76–1.69)	0.529	1.13 (0.59–2.16)	0.720	1.16 (0.60–2.25)	0.655

*Note:* Calculated using binary logistic regression. Crude is the unadjusted model. Model 1 was adjusted for sociodemographic variables (age, gender, race/ethnicity, body mass index, education level, marital status, family poverty income ratio, smoking status, drinking status, weekly physical activity time). Model 2 was adjusted for sociodemographic variables (age, gender, race/ethnicity, body mass index, education level, marital status, family poverty income ratio, smoking status, drinking status, weekly physical activity time), hypertension, diabetes, stroke, thyroid problem, and renal insufficiency.

Abbreviations: CI, confidence interval; OR, odds ratio.

### 3.3 Analysis of Non‐Linear Relationship

We used smoothed curve fitting (penalized spline method) to test whether there was a nonlinear relationship between UIC (a continuous variable) and low cognitive function. As shown in Figure S1, a linear relationship between UIC and low cognitive function was observed after fully adjusting for the covariates.

### 3.4 Subgroup Analysis

**FIGURE 2:**
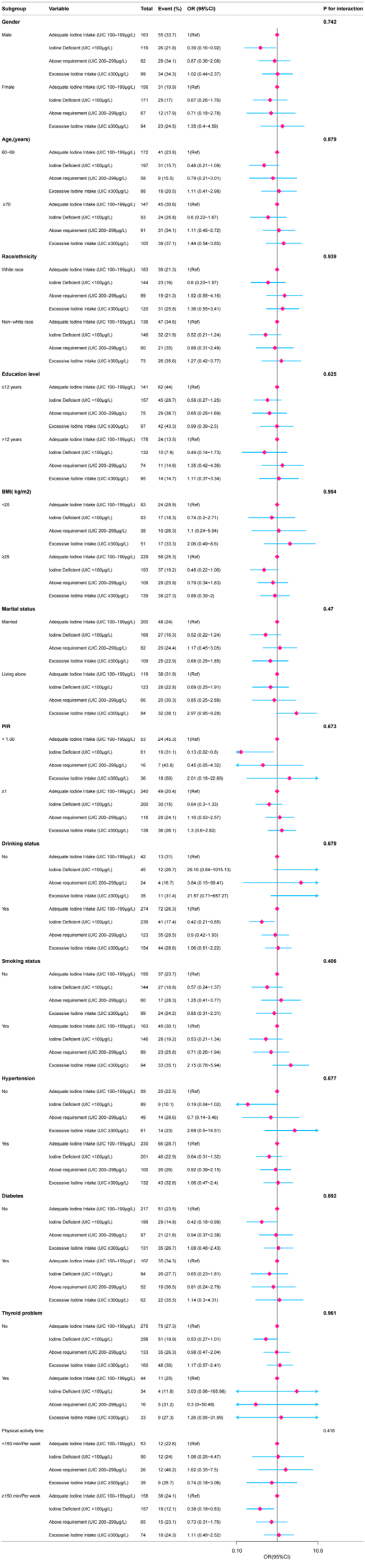
The relationship between urinary iodine concentration and cognitive function according to basic features. Except for the stratification component itself, each stratification factor was adjusted for all other variables (age, gender, race/ethnicity, body mass index, education level, marital status, family poverty income ratio, smoking status, alcohol consumption, hypertension, diabetes, stroke, thyroid problems, renal insufficiency, weekly physical activity time).

We performed stratified multivariate logistic regression and likelihood‐ratio interaction tests to assess whether the association between UIC and low cognitive function differed across subgroups. *p* for interaction was > 0.05 for all comparisons, indicating that the association remained consistent across most subgroups (including gender, age, race/ethnicity, education level, BMI, marital status, PIR, drinking status, smoking status, hypertension, diabetes, physical activity time, and thyroid problems) (Figure 2).

### 3.5 Sensitivity Analysis

The results of the sensitivity analyses excluding subjects classified as “never” drinkers are presented in Table S3. In the fully adjusted model, considering potential confounding factors, participants in the iodine deficiency group exhibited significantly lower odds of low cognitive performance compared to the adequate iodine intake group, with an adjusted OR of 0.42 (95% CI: 0.21–0.85; *p* = 0.016).

## 4 Discussion

The cross‐sectional study conducted on 951 elderly individuals revealed a significant association between UIC and cognitive performance. Although the normal range of urinary iodine for the elderly has not been definitively established, our findings suggest that iodine deficiency may serve as a protective factor against cognitive impairment in this population. After adjusting for potential confounding factors, individuals in the iodine deficient group had an adjusted OR of 0.54 (95% CI: 0.29–0.99; *p* = 0.046) for poor cognitive performance, compared to those with adequate iodine intake. Several sensitivity analyses confirmed the robustness of our results. Moreover, multiple reviews have highlighted that gaps persist in understanding the relationship between iodine status and cognitive function throughout life, particularly in older populations (Redman et al. 2016). By exploring the link between UIC and cognitive function in older adults, our study contributes to addressing this gap in the literature regarding the impact of iodine nutrition on cognitive health in this demographic.

Research exploring the link between UIC and cognitive function in older adults is scarce. In our review of the current literature, we identified a limited number of studies, with only one being directly comparable to our own. This study examined the relationship between iodized salt consumption and cognitive function in older Chinese adults and reported that a higher intake of iodized salt was associated with a lower risk of cognitive decline in both coastal and inland populations when compared to non‐iodized salt consumption (OR: 0.410; 95% CI, 0.351–0.480; *p* < 0.001) (Wu et al. 2023). Although previous studies have indicated that individuals consuming iodized salt tend to have higher UIC compared to those consuming non‐iodized salt, even in iodine‐replete regions such as coastal areas (Chen et al. 2017; Li et al. 2023), it is important to note that the study did not directly measure the UIC of the participants. Additionally, they employed different methods to assess cognitive function, which may explain the discrepancies between our findings and theirs. Interestingly, our results contrast with previous studies showing that iodine deficiency during pregnancy and in adolescence leads to impaired cognitive function and even cretinism in infants and adolescents (Bath et al. 2013; Santiago‐Fernandez et al. 2004). This discrepancy likely reflects differences in the study populations.

Iodine affects cognitive function mainly by regulating thyroid hormone synthesis and promoting cerebral white matter development (Redman et al. 2016), a mechanism that is particularly critical during early brain development stages such as fetal and school age (Ahmed et al. 2008; Delange 2001). Research has indicated that thyroid hormones play a crucial role in processes such as myelin production, cell migration and differentiation, synapse formation, dendritic growth, transcriptional regulation, and synaptic plasticity. These mechanisms, in turn, have varying effects on brain function, particularly influencing cognitive processes like learning and memory (Thompson and Potter 2000; Rivas and Naranjo 2007; Koibuchi 2008). White matter plays an important role in learning and cognitive and psychiatric disorders, involving mechanisms of nerve fibers and myelination (Fields 2008). However, myelin formation persists from late prenatal to late adolescence (Paus 2010), and white matter reaches a plateau of stability at approximately 30 years of age (Yeatman et al. 2014), suggesting that the brain structure is essentially mature in old age, so the effects of iodine deficiency on cognitive function in older adults are likely to be very limited. In older adults, subclinical thyroid dysfunction does not seem to significantly affect cognitive outcomes or dementia risk, while the role of overt thyroid dysfunction remains unclear (van Vliet et al. 2021). Notably, additional studies have shown that hyperthyroidism (both endogenous and exogenous) is associated with an increased risk of cognitive impairment in older adults (Papaleontiou and Brito 2023). These findings underscore the need for further investigation into the mechanisms linking iodine, thyroid hormones, and cognitive function across different age groups.

There are several advantages to our research. First, we directly assessed iodine nutritional status by measuring urinary iodine concentration. Second, while there are potential confounders, this study controlled for as many important confounding variables as possible in the statistical analyses. Finally, we conducted a sensitivity analysis to mitigate the impact of alcohol use on the results, and the findings showed that our results were stable. However, our study also has limitations. First, given the cross‐sectional design of the study, we were unable to establish a causal relationship between urinary iodine levels and cognitive decline. Further well‐designed prospective cohort studies are required to address this limitation. Second, the absence of thyroid hormone data from the NHANES 2013–2014 dataset introduces the possibility of residual confounding factors, such as undiagnosed thyroid dysfunction. However, we accounted for thyroid disease history as a covariate in our model, and the results remained consistent. Finally, while our cognitive assessments provide useful insights, they cannot substitute for a formal diagnosis based on clinical evaluation.

## 5 Conclusion

This cross‐sectional study provides the first evidence linking UIC to cognitive outcomes in older adults. Contrary to expectations, participants with iodine deficiency demonstrated significantly lower risk of cognitive decline compared to those with adequate iodine intake. These findings indicate a need to establish geriatric‐specific guidelines addressing age‐related metabolic adaptations, thereby bridging a critical gap in nutritional neuroscience. This suggests that iodine metabolic pathways may undergo age‐specific alterations in older adults, thus warranting prospective studies to validate the clinical validity of adjusting iodine nutritional thresholds in geriatric populations.

## Author Contributions

**Zeyong Sun:** conceptualization (lead), methodology (lead), writing – original draft (lead), writing – review and editing (lead). **Fengyang Song:** conceptualization (equal), methodology (equal), writing – original draft (equal), writing – review and editing (equal). **Xiaojuan Wei:** conceptualization (equal), methodology (equal), writing – original draft (equal), writing – review and editing (equal). **Wentan Huang:** supervision (supporting). **Lu Lu:** data curation (equal), software (supporting). **Kehua Liao:** writing – review and editing (supporting). **Shaozhou Mo:** investigation (supporting).

## Conflicts of Interest

The authors declare no conflicts of interest.

## Supporting information


**Figure S1:** Association between urinary iodine concentration and cognitive function odds ratio. Solid and dashed lines represent the predicted values and 95% confidence intervals. The analysis was adjusted for sociodemographic factors (age, gender, race/ethnicity, body mass index, education level, marital status, family poverty income ratio, smoking status, drinking status, and weekly physical activity time), as well as hypertension, diabetes, stroke, thyroid problems, and renal insufficiency. Only 99% of the data is shown.

**Table S1:** Baseline characteristics of the study population in crude data and five imputed datasets.

**Table S2:** Association of UIC and low cognitive performance Risk in five imputed datasets.

**Table S3:** Association of UIC and low cognitive function risk (participants Who Do Not Consume Alcohol Excluded).

## Data Availability

These survey data are free and publicly available, and can be downloaded directly from the NHANES website (http://www.cdc.gov/nchs/nhanes.htm) by users and researchers worldwide.
