# Supplementary material for: Association of Urinary Iodine Concentration With Cognitive Function Among Older Adults: NHANES 2011–2014
Source: Food Sci Nutr. 2025 Sep 3;13(9):e70906. doi: 10.1002/fsn3.70906 (PMC12406079; doi:10.1002/fsn3.70906)
Supplement: Supplementary file 1 — Figure S1: Association between urinary iodine concentration and cognitive function odds ratio. Solid and dashed lines represent the predicted values and 95% confidence intervals. The analysis was adjusted for sociodemographic factors (age, gender, race/ethnicity, body mass index, education level, marital status, family poverty income ratio, smoking status, drinking status, and weekly physical activity time), as well as hypertension, diabetes, stroke, thyroid problems, and renal insufficiency. Only 99% of the data is shown. [file FSN3-13-e70906-s004.docx]

eFigure 1


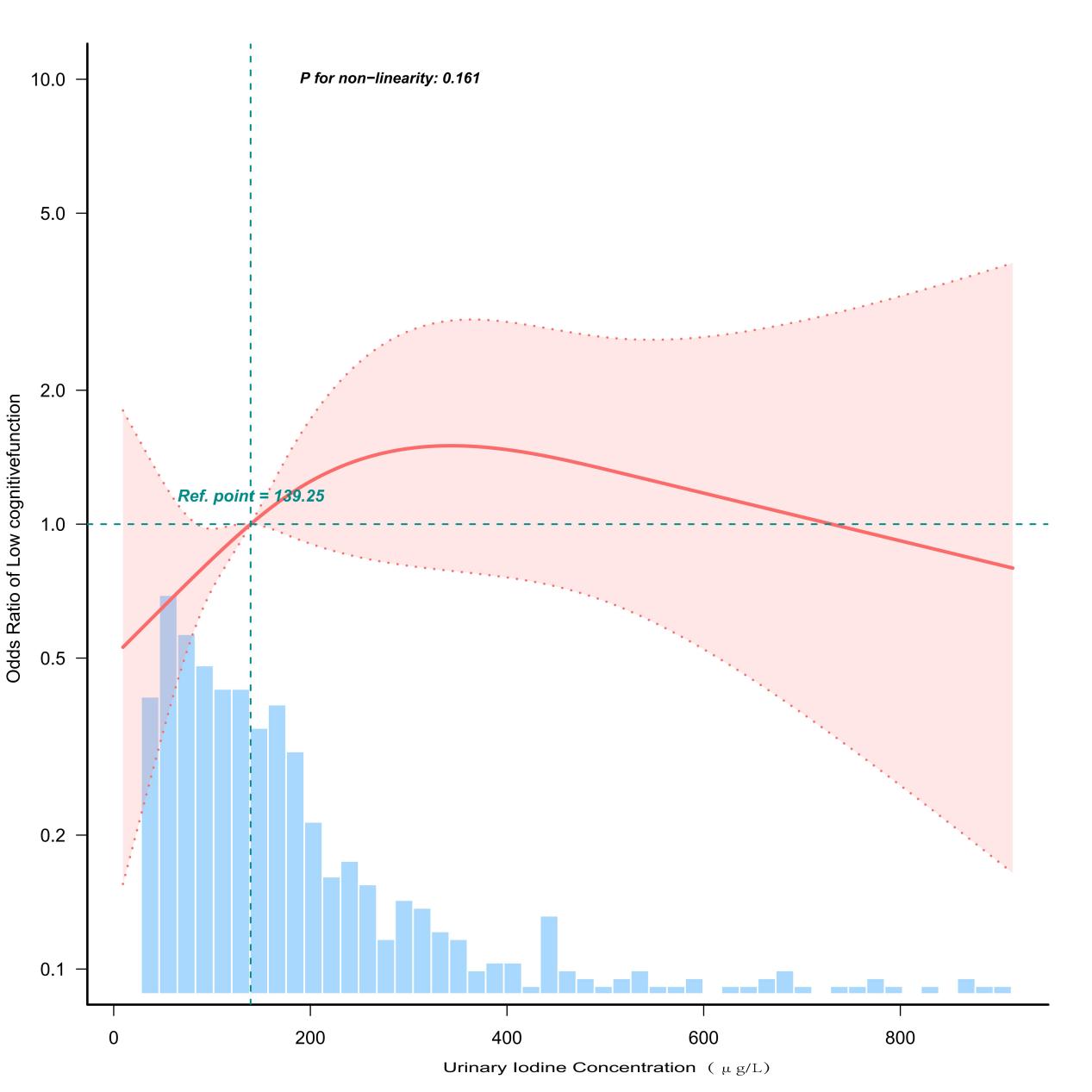


eFigure 1: Association between urinary iodine concentration and cognitive function odds ratio. Solid and dashed lines represent the predicted values and 95% confidence intervals. The analysis was adjusted for sociodemographic factors (age, gender, race/ethnicity, body mass index, education level, marital status, family poverty income ratio, smoking status, drinking status, and weekly physical activity time), as well as hypertension, diabetes, stroke, thyroid problems, and renal insufficiency. Only 99% of the data is shown.
