# Supplementary material for: Association of Urinary Iodine Concentration With Cognitive Function Among Older Adults: NHANES 2011–2014
Source: Food Sci Nutr. 2025 Sep 3;13(9):e70906. doi: 10.1002/fsn3.70906 (PMC12406079; doi:10.1002/fsn3.70906)
Supplement: Supplementary file 2 — Table S1: Baseline characteristics of the study population in crude data and five imputed datasets. [file FSN3-13-e70906-s002.docx]

**eTable 1 Baseline characteristics of the study population in crude data and five imputed datasets**

| Characteristics | Total  (n = 5706) | Crude  (n = 951) | Imputed Dataset 1  (n = 951) | Imputed Dataset 2  (n = 951) | Imputed Dataset 3  (n = 951) | Imputed Dataset 4  (n = 951) | Imputed Dataset 5  (n = 951) | *P*-value |
| --- | --- | --- | --- | --- | --- | --- | --- | --- |
| Age,n (%) |  |  |  |  |  |  |  | 1 |
| 60-69 | 3090 (54.2) | 515 (54.2) | 515 (54.2) | 515 (54.2) | 515 (54.2) | 515 (54.2) | 515 (54.2) |  |
| ≥70 | 2616 (45.8) | 436 (45.8) | 436 (45.8) | 436 (45.8) | 436 (45.8) | 436 (45.8) | 436 (45.8) |  |
| Gender, n (%) |  |  |  |  |  |  |  | 1 |
| Male | 2778 (48.7) | 463 (48.7) | 463 (48.7) | 463 (48.7) | 463 (48.7) | 463 (48.7) | 463 (48.7) |  |
| Female | 2928 (51.3) | 488 (51.3) | 488 (51.3) | 488 (51.3) | 488 (51.3) | 488 (51.3) | 488 (51.3) |  |
| Race/ethnicityn(%) |  |  |  |  |  |  |  | 1 |
| Non-Hispanic White | 2712 (47.5) | 452 (47.5) | 452 (47.5) | 452 (47.5) | 452 (47.5) | 452 (47.5) | 452 (47.5) |  |
| Non-Hispanic Black | 1350 (23.7) | 225 (23.7) | 225 (23.7) | 225 (23.7) | 225 (23.7) | 225 (23.7) | 225 (23.7) |  |
| Mexican American | 504 ( 8.8) | 84 (8.8) | 84 (8.8) | 84 (8.8) | 84 (8.8) | 84 (8.8) | 84 (8.8) |  |
| Other Hispanic | 552 ( 9.7) | 92 (9.7) | 92 (9.7) | 92 (9.7) | 92 (9.7) | 92 (9.7) | 92 (9.7) |  |
| Other Race-including Multi-Racial | 588 (10.3) | 98 (10.3) | 98 (10.3) | 98 (10.3) | 98 (10.3) | 98 (10.3) | 98 (10.3) |  |
| BMI, n (%) |  |  |  |  |  |  |  | 1 |
| <25 kg/m² | 1622 (28.5) | 265 (28.4) | 273 (28.7) | 269 (28.3) | 272 (28.6) | 274 (28.8) | 269 (28.3) |  |
| 25 <30 kg/m² | 2022 (35.5) | 333 (35.7) | 337 (35.4) | 338 (35.5) | 340 (35.8) | 337 (35.4) | 337 (35.4) |  |
| ≥30 kg/m² | 2045 (35.9) | 336 (36) | 341 (35.9) | 344 (36.2) | 339 (35.6) | 340 (35.8) | 345 (36.3) |  |
| Education level, n (%) |  |  |  |  |  |  |  | 1 |
| Low Educational Level | 2826 (49.5) | 470 (49.5) | 471 (49.5) | 471 (49.5) | 472 (49.6) | 471 (49.5) | 471 (49.5) |  |
| Intermediate Educational Level | 1618 (28.4) | 269 (28.3) | 270 (28.4) | 270 (28.4) | 269 (28.3) | 270 (28.4) | 270 (28.4) |  |
| High Educational Leve | 1260 (22.1) | 210 (22.1) | 210 (22.1) | 210 (22.1) | 210 (22.1) | 210 (22.1) | 210 (22.1) |  |
| Marry status, n (%) |  |  |  |  |  |  |  | 1 |
| Married | 3203 (56.2) | 533 (56.2) | 534 (56.2) | 534 (56.2) | 533 (56) | 534 (56.2) | 535 (56.3) |  |
| Never married | 336 ( 5.9) | 56 (5.9) | 56 (5.9) | 56 (5.9) | 56 (5.9) | 56 (5.9) | 56 (5.9) |  |
| Living with partner | 144 ( 2.5) | 24 (2.5) | 24 (2.5) | 24 (2.5) | 24 (2.5) | 24 (2.5) | 24 (2.5) |  |
| widowed, divorced, or separated individuals | 2021 (35.4) | 336 (35.4) | 337 (35.4) | 337 (35.4) | 338 (35.5) | 337 (35.4) | 336 (35.3) |  |
| PIR, n (%) |  |  |  |  |  |  |  | 0.995 |
| <1 | 1117 (19.9) | 166 (19.3) | 193 (20.3) | 188 (19.8) | 191 (20.1) | 192 (20.2) | 187 (19.7) |  |
| ≥1 | 4498 (80.1) | 694 (80.7) | 758 (79.7) | 763 (80.2) | 760 (79.9) | 759 (79.8) | 764 (80.3) |  |
| Smoking status, n (%) |  |  |  |  |  |  |  | 1 |
| No | 2754 (48.3) | 459 (48.3) | 459 (48.3) | 459 (48.3) | 459 (48.3) | 459 (48.3) | 459 (48.3) |  |
| Yes | 2952 (51.7) | 492 (51.7) | 492 (51.7) | 492 (51.7) | 492 (51.7) | 492 (51.7) | 492 (51.7) |  |

**eTable 1.Continued**

| Characteristics | Total  (n = 5706) | Crude  (n = 951) | Imputed Dataset 1  (n = 951) | Imputed Dataset 2  (n = 951) | Imputed Dataset 3  (n = 951) | Imputed Dataset 4  (n = 951) | Imputed Dataset 5  (n = 951) | *P*-value |  |
| --- | --- | --- | --- | --- | --- | --- | --- | --- | --- |
| Drinking status n (%) |  |  |  |  |  |  |  | 1 |  |
| never | 890 (15.6) | 146 (15.6) | 148 (15.6) | 147 (15.5) | 150 (15.8) | 148 (15.6) | 151 (15.9) |  |  |
| former | 1548 (27.2) | 254 (27.2) | 259 (27.2) | 260 (27.3) | 259 (27.2) | 258 (27.1) | 258 (27.1) |  |  |
| mild | 2314 (40.7) | 380 (40.7) | 387 (40.7) | 387 (40.7) | 387 (40.7) | 388 (40.8) | 385 (40.5) |  |  |
| moderate | 526 ( 9.2) | 86 (9.2) | 87 (9.1) | 90 (9.5) | 86 (9) | 89 (9.4) | 88 (9.3) |  | |
| heavy | 410 ( 7.2) | 67 (7.2) | 70 (7.4) | 67 (7) | 69 (7.3) | 68 (7.2) | 69 (7.3) |  | |
| Hypertension, n (%) |  |  |  |  |  |  |  | 1 | |
| No | 1728 (30.3) | 288 (30.3) | 288 (30.3) | 288 (30.3) | 288 (30.3) | 288 (30.3) | 288 (30.3) |  | |
| Yes | 3978 (69.7) | 663 (69.7) | 663 (69.7) | 663 (69.7) | 663 (69.7) | 663 (69.7) | 663 (69.7) |  | |
| Diabetes, n (%) |  |  |  |  |  |  |  | 1 | |
| No | 3846 (67.4) | 641 (67.4) | 641 (67.4) | 641 (67.4) | 641 (67.4) | 641 (67.4) | 641 (67.4) |  | |
| Yes | 1860 (32.6) | 310 (32.6) | 310 (32.6) | 310 (32.6) | 310 (32.6) | 310 (32.6) | 310 (32.6) |  | |
| Stroke, n (%) |  |  |  |  |  |  |  | 1 | |
| No | 5381 (94.3) | 896 (94.3) | 897 (94.3) | 897 (94.3) | 897 (94.3) | 897 (94.3) | 897 (94.3) |  | |
| Yes | 324 ( 5.7) | 54 (5.7) | 54 (5.7) | 54 (5.7) | 54 (5.7) | 54 (5.7) | 54 (5.7) |  | |
| Renal insufficiency, n (%) |  |  |  |  |  |  |  | 1 | |
| No | 5355 (93.9) | 891 (93.9) | 893 (93.9) | 893 (93.9) | 893 (93.9) | 893 (93.9) | 892 (93.8) |  | |
| Yes | 349 ( 6.1) | 58 (6.1) | 58 (6.1) | 58 (6.1) | 58 (6.1) | 58 (6.1) | 59 (6.2) |  | |
| Thyroid problem |  |  |  |  |  |  |  | 1 | |
| No | 4944 (86.6) | 824 (86.6) | 824 (86.6) | 824 (86.6) | 824 (86.6) | 824 (86.6) | 824 (86.6) |  | |
| Yes | 762 (13.4) | 127 (13.4) | 127 (13.4) | 127 (13.4) | 127 (13.4) | 127 (13.4) | 127 (13.4) |  | |
| WPAT,  Median (IQR) | 300.0  (125.0, 750.0) | 320.0  (140.0, 780.0) | 300.0  (120.0, 720.0) | 300.0  (135.0, 720.0) | 300.0  (120.0,690.0) | 300.0  (120.0, 750.0) | 350.0  (140.0, 840.0) | 0.213 | |

Notes: PIR=poverty income ratio; BMI= body mass index ;WPAT=Weekly Physical Activity Time
