# Supplementary material for: Association of Urinary Iodine Concentration With Cognitive Function Among Older Adults: NHANES 2011–2014
Source: Food Sci Nutr. 2025 Sep 3;13(9):e70906. doi: 10.1002/fsn3.70906 (PMC12406079; doi:10.1002/fsn3.70906)
Supplement: Supplementary file 3 — Table S2: Association of UIC and low cognitive performance Risk in five imputed datasets. [file FSN3-13-e70906-s003.docx]

**eTable 2. Association of UIC and low cognitive performance Risk in five imputed datasets**

| **U I C (µg/L)** | **Cases/Participants** | **crude.OR (95%CI)** | ***P*_value** | **adj.OR (95%CI)** | ***P*_value** |
| --- | --- | --- | --- | --- | --- |
| First imputed dataset |  |  |  |  |  |
| Iodine Deficient (<100) | 55/290(19.0) | 0.63 (0.43~0.93) | 0.020 | 0.53 (0.34~0.82) | 0.004 |
| Adequate Iodine Intake (100~199) | 86/319(27.0) | 1(Ref) |  | 1(Ref) |  |
| Above requirement (200~299) | 40/149(26.8) | 0.99 (0.64~1.54) | 0.979 | 0.76 (0.46~1.26) | 0.292 |
| Excessive Iodine Intake (≥300) | 57/193(29.5) | 1.14 (0.76~1.69) | 0.529 | 0.96 (0.61~1.51) | 0.848 |
| Second imputed dataset |  |  |  |  |  |
| Iodine Deficient (<100) | 55/290(19.0) | 0.63 (0.43~0.93) | 0.020 | 0.53 (0.34~0.83) | 0.005 |
| Adequate Iodine Intake (100~199) | 86/319(27.0) | 1(Ref) |  | 1(Ref) |  |
| Above requirement (200~299) | 40/149(26.8) | 0.99 (0.64~1.54) | 0.979 | 0.77 (0.47~1.28) | 0.316 |
| Excessive Iodine Intake (≥300) | 57/193(29.5) | 1.14 (0.76~1.69) | 0.529 | 0.98 (0.62~1.54) | 0.925 |
| Third imputed dataset |  |  |  |  |  |
| Iodine Deficient (<100) | 55/290(19) | 0.63 (0.43~0.93) | 0.020 | 0.51 (0.33~0.79) | 0.003 |
| Adequate Iodine Intake (100~199) | 126/468(26.9) | 1(Ref) |  | 1(Ref) |  |
| Above requirement (200~299) | 25/75(33.3) | 0.99 (0.64~1.54) | 0.979 | 0.75 (0.46~1.25) | 0.271 |
| Excessive Iodine Intake (≥300) | 32/118(27.1) | 1.14 (0.76~1.69) | 0.529 | 0.92 (0.58~1.46) | 0.726 |
| fourth imputed dataset |  |  |  |  |  |
| Iodine Deficient (<100) | 55/290(19) | 0.63 (0.43~0.93) | 0.020 | 0.53 (0.34~0.82) | 0.005 |
| Adequate Iodine Intake (100~199) | 126/468(26.9) | 1(Ref) |  | 1(Ref) |  |
| Above requirement (200~299) | 25/75(33.3) | 0.99 (0.64~1.54) | 0.979 | 0.77 (0.47~1.28) | 0.319 |
| Excessive Iodine Intake (≥300) | 32/118(27.1) | 1.14 (0.76~1.69) | 0.529 | 0.96 (0.61~1.52) | 0.873 |
| fifth imputed dataset |  |  |  |  |  |
| Iodine Deficient (<100) | 55/290(19) | 0.63 (0.43~0.93) | 0.020 | 0.52 (0.34~0.82) | 0.004 |
| Adequate Iodine Intake (100~199) | 126/468(26.9) | 1(Ref) |  | 1(Ref) |  |
| Above requirement (200~299) | 25/75(33.3) | 0.99 (0.64~1.54) | 0.979 | 0.77 (0.47~1.27) | 0.307 |
| Excessive Iodine Intake (≥300) | 32/118(27.1) | 1.14 (0.76~1.69) | 0.529 | 0.95 (0.61~1.5) | 0.838 |

Note: Calculated using binary logistic regression. UIC=Urinary Iodine Concentration; OR=odds ratio; CI= confidence interval. Crude is the unadjusted model. Adjusted for sociodemographic factors ( age, gender, race/ethnicity, body mass index, education level, marital status, family poverty income ratio, smoking status, drinking status, weekly physical activity time), hypertension, diabetes, stroke, thyroid problem, renal insufficiency.
