# Supplementary material for: Association of Urinary Iodine Concentration With Cognitive Function Among Older Adults: NHANES 2011–2014
Source: Food Sci Nutr. 2025 Sep 3;13(9):e70906. doi: 10.1002/fsn3.70906 (PMC12406079; doi:10.1002/fsn3.70906)
Supplement: Supplementary file 4 — Table S3: Association of UIC and low cognitive function risk (participants Who Do Not Consume Alcohol Excluded). [file FSN3-13-e70906-s001.docx]

**eTable 3. Association of UIC and low Cognitive function Risk (Participants Who Do Not Consume Alcohol Excluded)**

| Urinary Iodine Concentration (µg/L) | Cases/Participants | crude.OR (95%CI) | *P*_value | adj.OR (95%CI) | *P*_value |
| --- | --- | --- | --- | --- | --- |
| Iodine Deficient (<100) | 41/236(17.4) | 0.59 (0.38~0.91) | 0.016 | 0.42 (0.21~0.85) | 0.016 |
| Adequate Iodine Intake (100~199) | 72/274(26.3) | 1(Ref) |  | 1(Ref) |  |
| Above requirement (200~299) | 35/123(28.5) | 1.12 (0.69~1.79) | 0.651 | 0.90 (0.42~1.93) | 0.784 |
| Excessive Iodine Intake (≥300) | 44/154(28.6) | 1.12 (0.72~1.74) | 0.608 | 1.06 (0.51~2.22) | 0.878 |

Note: Calculated using binary logistic regression. UIC=Urinary Iodine Concentration; OR=odds ratio; CI= confidence interval. Crude is the unadjusted model. Adjusted for sociodemographic factors (age, gender, race/ethnicity, body mass index, education level, marital status, family poverty income ratio, smoking status, drinking status, weekly physical activity time), as well as hypertension, diabetes, stroke, thyroid problem,and renal insufficiency.
